# Czochralski growth of mixed cubic sesquioxide crystals in the ternary system Lu_2_O_3_–Sc_2_O_3_–Y_2_O_3_

**DOI:** 10.1107/S2052520621005321

**Published:** 2021-07-15

**Authors:** Christian Kränkel, Anastasia Uvarova, Émile Haurat, Lena Hülshoff, Mario Brützam, Christo Guguschev, Sascha Kalusniak, Detlef Klimm

**Affiliations:** a Leibniz-Institut für Kristallzüchtung (IKZ), Max-Born-Str. 2, 12489 Berlin, Germany; b Service de Recherches de Métallurgie Physique, Université Paris-Saclay, CEA, 3 rue Joliot Curie, Gif-sur-Yvette, 91190, France

**Keywords:** crystal growth, optical materials, phase diagrams, melting points, rare earth sesquioxides

## Abstract

Cubic rare-earth sesquioxide crystals are strongly demanded host materials for high power lasers, but due to their high melting points investigations on their thermodynamics and the growth of large-size crystals of high optical quality remain a challenge. Detailed thermal investigations of the ternary system Lu_2_O_3_–Sc_2_O_3_–Y_2_O_3_ revealing a large range of compositions with melting temperatures below 2200°C and a minimum of 2053°C for the composition (Sc_0.45_Y_0.55_)_2_O_3_ are presented. These reduced temperatures enable for the first time the growth of high optical quality mixed sesquioxide crystals with disordered structure by the conventional Czochralski method from iridium crucibles. An (Er­_0.07_Sc_0.50_Y_0.43_)_2_O_3_ crystal is successfully grown and characterized with respect to its crystallographic properties as well as its composition, thermal conductivity and optical absorption in the 1 µm range.

## Abstract 2

The phase diagram of the ternary system Lu_2_O_3_–Sc_2_O_3_–Y_2_O_3_ is investigated and compositions with melting points below 2200°C are found. This allows for the first successful growth of the mixed cubic sesquioxide crystal (Er_0.07_Sc_0.50_Y_0.43_)_2_O_3_ by the Czochralski method from an iridium crucible.

## 1. Introduction   

Cubic rare-earth sesquioxide crystals of the form RE_2_O_3_ with RE = Lu, Sc or Y are excellent host materials for high power and ultrafast lasers (Kränkel, 2015 ▸). In particular when doped with Yb^3+^ these materials exhibit outstanding laser performance in high-power continuous wave (Weichelt *et al.*, 2012 ▸; Peters *et al.*, 2011 ▸) and ultrafast (Marchese *et al.*, 2007 ▸; Baer *et al.*, 2010 ▸) operation in the 1 µm range. Also when doped with Ho^3+^ or Tm^3+^ efficient performance was obtained in the 2 µm spectral range (Koopmann, Peters *et al.*, 2011 ▸; Koopmann, Lamrini *et al.*, 2011*a* ▸,*b* ▸; Koopman *et al.*, 2013 ▸; Loiko *et al.*, 2018 ▸; Lagatsky *et al.*, 2012 ▸). Finally, Er^3+^-doped sesquioxide crystals are among the most efficient materials for the generation of 3 µm radiation (Li *et al.*, 2012 ▸; Fan *et al.*, 2016 ▸). This success is enabled by a unique interplay between outstanding thermo-mechanical properties (Peters *et al.*, 2011 ▸) and a strong crystal field (Dorenbos, 2000 ▸), which homogeneously broadens the emission lines and shifts the peak wavelengths to unusual, often longer wavelengths compared to other host materials (von Brunn *et al.*, 2016 ▸).

**Figure 1:**
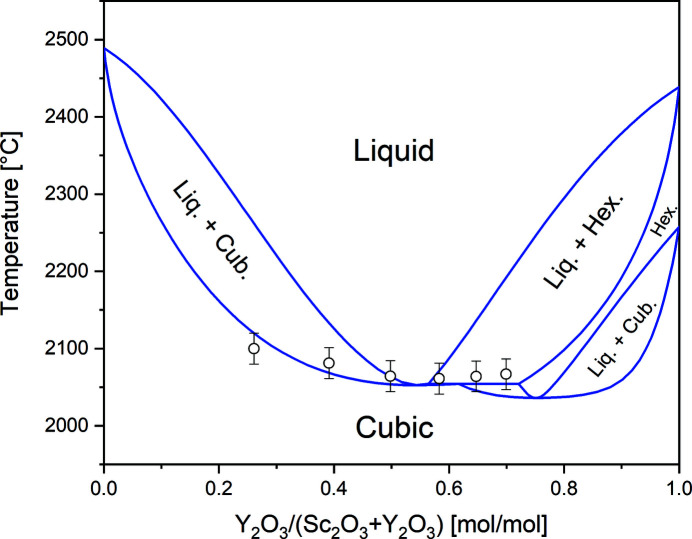
Refined binary phase diagram of the system Sc_2_O_3_–Y_2_O_3_. Open circles refer to solidus points experimentally obtained in the DTA measurements.

Cubic sesquioxides form complete solution series, allowing them to be mixed in arbitrary ratios. For the largest area of the corresponding ternary phase diagram Lu_2_O_3_–Sc_2_O_3_–Y_2_O_3_, the melt crystallizes directly in a cubic phase with a disordered structure caused by a random distribution of Lu, Sc and Y on the cation sites. Only very Y_2_O_3_-rich phases initially crystallize in a high-temperature hexagonal phase (see Fig. 1 ▸), while their room-temperature phase is cubic, too. In mixed cubic phase sesquioxide crystals of the form (Lu_*x*_Sc_*y*_Y_*z*_)_2_O_3_ with *x* + *y* + *z* = 1, even larger emission bandwidths than in pure sesquioxides are observed (Beil *et al.*, 2013 ▸).

As early as 1975 the mixed sesquioxide Nd:YScO_3_ was investigated as a laser material (Bagdasarov *et al.*, 2013 ▸), but the main advantage of the corresponding disordered structure was revealed much later utilizing Yb:LuScO_3_. In this material, the emission bandwidths of the constituents Yb:Lu_2_O_3_ and Yb:Sc_2_O_3_ are combined, resulting in an inhomogeneously broadened emission band of more than 20 nm in width (Baer *et al.*, 2009 ▸; Saraceno *et al.*, 2011 ▸; Südmeyer *et al.*, 2009 ▸). In experiments with Nd:(Lu_1–*x*_Sc_*x*_)_2_O_3_, compositional tuning enabled a fine-tuning of the laser emission wavelength to a desired value (Reichert *et al.*, 2012 ▸). As a drawback, a disordered structure leads to a reduced thermal conductivity. However, the reported values are still higher than for other materials with comparable emission bandwidth (Beil *et al.*, 2013 ▸). This holds also for Tm^3+^-doped mixed sesquioxides (Stevenson *et al.*, 2018 ▸).

Unfortunately, the growth of sesquioxide crystals is very challenging. Lu_2_O_3_, ScO_3_ and Y_2_O_3_ possess melting points well in excess of 2350°C (Bale *et al.*, 2016 ▸; Kenisarin & Chekhovskoy, 1975 ▸; Navrotsky *et al.*, 2005 ▸). Iridium, the most common crucible material for the growth of high-melting-point oxide crystals, is not suitable for such high growth temperatures due to a loss of structural integrity at temperatures close to its melting point of 2410°C. Therefore, crucible-free growth methods have been extensively investigated for the growth of sesquioxide single crystals. These included the Verneuil method (Barta *et al.*, 1958 ▸), laser-heated pedestal growth (Tissue *et al.*, 1991 ▸) and pulsed laser deposition (Prentice *et al.*, 2018 ▸). More recently, significant progress was made by the growth from the flux (Veber *et al.*, 2011 ▸; Druon *et al.*, 2013 ▸; Velázquez *et al.*, 2015 ▸) and by the optical floating zone technique (Gasson & Cockayne, 1970 ▸; Uvarorova *et al.*, 2019 ▸; Liu *et al.*, 2020 ▸; Chen *et al.*, 2020 ▸; Liu *et al.*, 2019 ▸). However, all of these methods resulted in crystals with limited dimensions, purity, and/or quality.

Due to the difficulties in the growth from the melt, the fabrication of ceramic sesquioxides was also investigated. Ceramic optical materials are fabricated by sintering at temperatures well below their melting points. This approach is particularly suited to materials with a cubic crystal symmetry such as the rare-earth sesquioxides investigated here. The first laser demonstration in Nd:Y_2_O_3_ ceramics dates back to 2001 (Lu *et al.*, 2001 ▸). Since then, considerable progress has been made in the fabrication and laser operation of ceramic sesquioxides doped with Yb^3+^ (Tokurakawa *et al.*, 2009 ▸), Tm^3+^ (Lagatsky, Antipov *et al.*, 2012 ▸) or Er^3+^ (Uehara *et al.*, 2018 ▸). Recently, the fabrication (Lu *et al.*, 2013 ▸; Liu *et al.*, 2015 ▸; Thoř *et al.*, 2021 ▸) and laser operation (Zhou *et al.*, 2017 ▸; Jing *et al.*, 2018 ▸; Hao *et al.*, 2018 ▸; Toci *et al.*, 2021 ▸) of mixed sesquioxides has drawn the attention of researchers. It was known that mixed sesquioxide ceramics provide ultrashort pulses in mode-locked operation (Zhao *et al.*, 2020 ▸). Although high quality sesquioxide ceramics can be synthesized, their fabrication process is rather complicated, time consuming and often includes the use of foreign sintering aids such as ZrO_2_ or LiF. Moreover, ceramics are prone to increased scattering at grain boundaries, pores and secondary phases introduced by sintering aids (Xiao *et al.*, 2020 ▸).

Rhenium is the only crucible material mechanically stable and inert to the melt in the required growth atmospheres. Therefore, the most successful growth results of cubic sesquioxides up to now were obtained using rhenium crucibles. However, the fabrication of Re crucibles is difficult and costly. Its hardness hinders the fabrication of crucibles from metal blocks. Powder metallurgic Re crucibles made of pressed and sintered Re powder often remain porous and exhibit leakage. Only crucibles made by expensive and time-consuming galvanic methods exhibit properties suitable for the reproducible and reliable growth of sesquioxide crystals. However, all rhenium crucibles remain sensitive to oxygen in the growth atmosphere and the window between an oxygen partial pressure low enough to avoid corrosion of the crucible and still sufficient to grow high quality oxide crystals is narrow (Peters *et al.*, 2008 ▸). In addition, insulation materials withstanding such high growth are required. Zirconia felts have been shown to be suitable, but these are expensive and need to be replaced frequently.

Past growth attempts using rhenium crucibles included the micro-pulling down method (Mun *et al.*, 2007 ▸) and the edge-defined film-fed growth method (Yin *et al.*, 2020 ▸). Also the growth by the Czochralski method, the most established growth method for high-quality single crystals, has been investigated in detail. At the high temperatures required for the Czochralski growth of cubic sesquioxides, the average photon energy of the emitted heat radiation is increased and at the same time the band gap energy of the growing crystal is reduced due to the increasing atomic vibration (Varshni, 1967 ▸). Thus, growing sesquioxide crystals partially absorb the thermal radiation and exhibit a reduced heat transport through the crystal. This leads to significant growth instabilities and consequently very short crystals limited in quality (Fornasiero *et al.*, 1999 ▸, 2000 ▸; Petermann *et al.*, 2002 ▸). The best results up to now, including cm^3^-scale single crystals of highest laser quality, were obtained utilizing the heat-exchanger method (Peters *et al.*, 2020 ▸). Nevertheless, the significant costs for crucible and insulation as well as the increased risk for crucible damage hindered commercial success of this growth approach up to now. The growth of high-quality cubic yttria crystals is further impeded by the existence of a hexagonal high-temperature phase mentioned previously. The transition to the cubic phase taking place at temperatures around 2280°C (Zinkevich, 2007 ▸; Tsukuda, 1988 ▸), and thus below the melting point, is suppressed in the solidified medium. The high mobility of the atoms at such high temperatures enables a reorganization even to macroscopic cubic phase grains, but still defects, grain boundaries and scattering centers are typically found in cubic yttria, resulting in an inferior optical quality.

In this work, we re-investigate the binary and ternary phase diagrams of the system Lu_2_O_3_–Sc_2_O_3_–Y_2_O_3_ to identify compositions with reduced melting points suitable for growth by the conventional Czochralski method from standard iridium crucibles. We also aim to explore the limits of yttria concentration in such compositions as this component is significantly cheaper than lutetia or scandia. In our thermodynamic investigations, we experimentally confirm liquidus temperatures below 2100°C in the system Sc_2_O_3_–Y_2_O_3_, which are further reduced to below 2050°C by the admixture of the laser-active Er_2_O_3_. The range of compositions with melting temperatures suitable for the growth from iridium crucibles covers lattice parameters between 10.1 Å and 10.4 Å. This allows for lattice-matched cubic substrates for epitaxial film growth as well as a wide crystal field tuning of the emission bands of rare-earth-doped sesquioxide laser gain materials. In a proof-of-principle experiment we successfully grow a high-quality mixed sesquioxide boule by the Czochralski method for the first time. This crystal has an Er-doping concentration of 7 at.% with respect to the cation sites, *i.e.* a composition of (Er_0.07_Sc_0.50_Y_0.43_)_2_O_3_, and exhibits large single crystalline areas. We characterize it with respect to its crystallographic, thermal and spectroscopic properties.

## 2. Thermodynamic investigations   

For the determination of the melting temperatures we used a simultaneous thermal analyzer (NETZSCH STA429C) enabling differential thermal analysis (DTA) measurements up to temperatures of about 2400°C. Tens of milligrams of the starting composition were put in tungsten crucibles placed on a sample holder with tungsten–rhenium thermocouples. The crucibles were covered by a tungsten lid to reduce heat exchange by thermal radiation, which proved to be beneficial for a higher sensitivity of the thermoanalytic setup. To avoid oxidation of the tungsten crucible, the measurement chamber was evacuated to < 10^−5^ mbar and flooded with helium (99.9999% purity) three times. The measurements were performed at ambient pressure under He atmosphere. After the measurement of each sample, the composition was altered by additional weighing of one component into the crucible. To account for the known change of the characteristic function of the tungsten–rhenium thermocouples due to the preferred evaporation of rhenium at such high temperatures (Burns & Scroger, 1989 ▸), they were regularly recalibrated against pure Al_2_O_3_ with a melting point of 2054°C (Belmonte *et al.*, 2013 ▸). In the first cycle, each composition was heated to temperatures above the melting point, which was found to be below 2250°C for all compositions under investigation, and held at the maximum temperature for about 10 min for homogenization. Successively, the sample was cooled to 1500°C and reheated to ∼2250°C three times to receive three independent DTA curves before it was cooled down to room temperature for re-charging of the crucible.

First, we re-investigated the binary phase diagram of the Sc_2_O_3_–Y_2_O_3_ system employing DTA measurements with six different powder compositions between (Sc_0.30_Y_0.70_)_2_O_3_ and (Sc_0.74_Y_0.26_)_2_O_3_. Compared to literature data for this system (Badie, 1978 ▸), we found the solidus temperatures to be about 70 K lower, while the azeotrope point estimated to be at (Sc_0.45_Y_0.55_)_2_O_3_ is very close to the value reported by Badie (1978 ▸). For Y_2_O_3_ ratios below 55% only the cubic solid phase exists, but for higher yttrium content, we clearly identified the existence of the high-temperature hexagonal phase, which is known to reduce the structural quality of crystals in the cubic space group 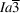 (bixbyite structure) at room temperature. For compositions between (Sc_0.68_Y_0.32_)_2_O_3_ and (Sc_0.30_Y_0.70_)_2_O_3_ we estimate the liquidus temperature to be below 2200°C and thus in the range accessible with iridium crucibles. Fig. 1 ▸ shows a refined phase diagram for the binary system Sc_2_O_3_–Y_2_O_3_. The assessment was made using *FactSage* (GTT Technologies, 2020 ▸) software and also takes into account all data for the ternary system presented later. It should be noted that not taking into account the data for the ternary system, an even better overlap of simulation and measurement is possible for the binary system. Due to the limitation of our DTA setup we were not able to measure the melting points of the pure sesquioxides and rely on values provided by the *FactSage* database (Bale *et al.*, 2016 ▸) used for the calculation of all phase diagrams in this work. While our results and other literature values (Navrotsky *et al.*, 2005 ▸) indicate that the values given by Bale *et al.* (2016 ▸) might be too high, in the area of interest for this work, *i.e.* compositions with melting points below 2200°C, we regard our results to be very reliable.

**Figure 2:**
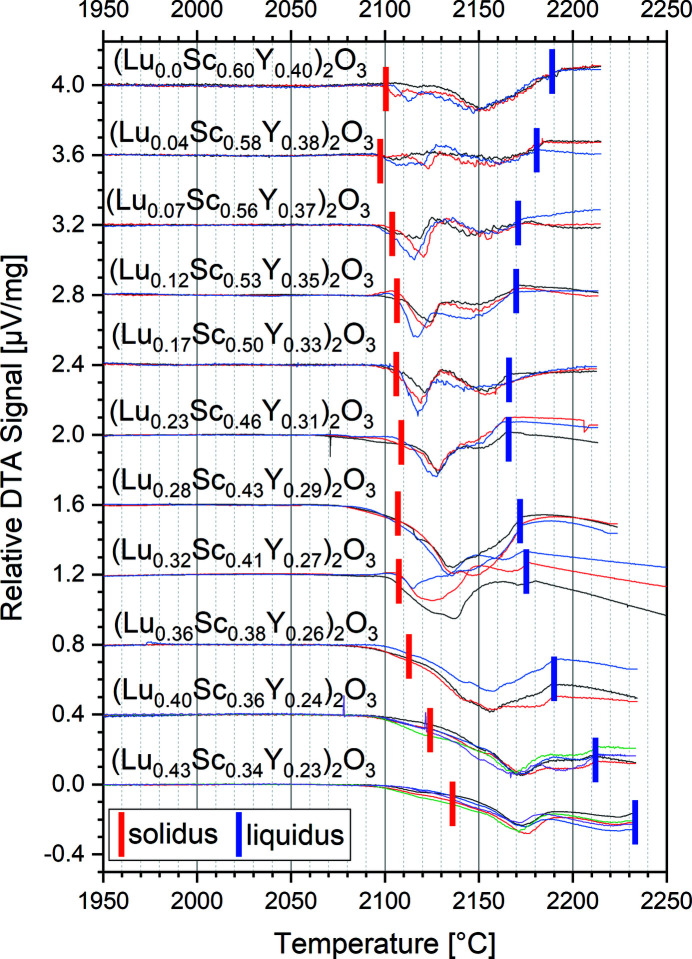
DTA curves for different compositions of the Lu_2_O_3_–Sc_2_O_3_–Y_2_O_3_ ternary system. The black, red and blue curves represent the first, second and third measurement, respectively, for each sample. For clarity, an offset decreasing in intervals of 0.4 µV mg^−1^ from 4 µV mg^−1^ at 0%Lu to 0 µV mg^−1^ at 43%Lu was added to the data.

**Figure 3:**
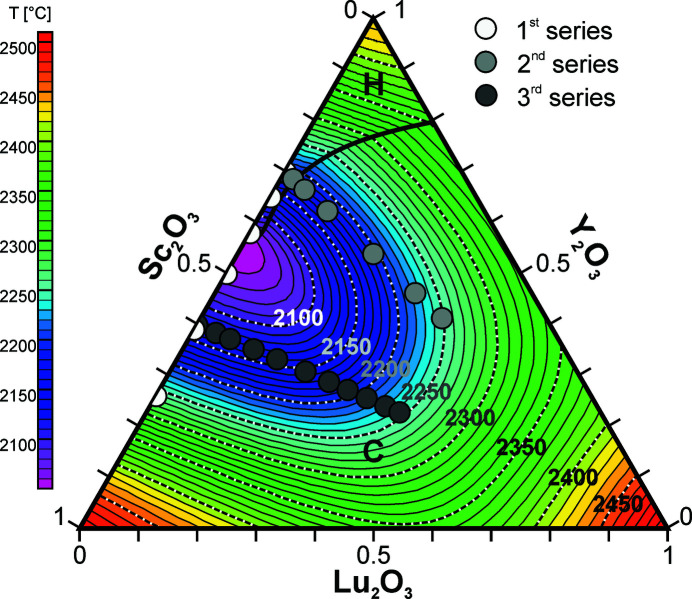
Phase diagram of the Lu_2_O_3_–Sc_2_O_3_–Y_2_O_3_ ternary system (projection on the liquidus surface). Isotherms are separated by 10°C. Grey circles indicate the compositions for which DTA measurements were performed. The hexagonal high-temperature phase of yttria is marked with H and C indicates the dominating cubic bixbyite phase.

Further measurements for selected compositions within the ternary phase diagram Lu_2_O_3_–Sc_2_O_3_–Y_2_O_3_ were conducted to narrow down the range of ternary compositions with melting points below 2200°C. Fig. 2 ▸ shows the result of a DTA measurement series as an example. We started with the composition (Lu_0.0_Sc_0.6_Y_0.4_)_2_O_3_ shown in the top and successively added small amounts of lutetia powder to the sample up to the final composition (Lu_0.43_Sc_0.34_Y_0.23_)_2_O_3_ in the bottom of Fig. 2 ▸. While it is not the aim of this article to explain all features of these curves, it can clearly be seen that for all curves an endothermic reaction, *i.e.* a decrease of the curve, indicates the start of the phase transition (marked with red bars in Fig. 2 ▸) from the solid into the liquid phase at temperatures below 2150°C. For all compositions except the two with the highest Lu content the peak related to the phase transition ends at temperatures clearly below 2200°C (marked with blue bars in Fig. 2 ▸). It should be noted that a DTA peak, even for congruently melting samples, always has a finite width. This width results from a limited thermal transport between the sample and the DTA sensor. For the given experimental conditions this width is in the order of 30 to 40 K. Hence, the liquidus temperatures are even expected to be ∼30 to 40 K below the blue bars marking the end of the melting peaks in Fig. 2 ▸. From these DTA curves it is obvious that the melting process for all samples with Lu concentrations between 0 and ∼0.3 begins at about 2100°C. This means that the solidus of the ternary system is very flat in this range, and rises significantly for Lu concentrations in excess of 0.36. In the ternary phase diagram shown in Fig. 3 ▸ the compositions investigated in Fig. 2 ▸ are labeled ‘3rd series’ and indicated by dark gray circles. In another series we investigated compositions starting at (Lu_0.02_Sc_0.29_Y_0.69_)_2_O_3_ and ending at (Lu_0.41_Sc_0.18_Y_0.41_)_2_O_3_ by again successively adding lutetia. These compositions are indicated by light gray and those for the Sc_2_O_3_–Y_2_O_3_ binary system by white circles in Fig. 3 ▸.

Based on all three series of measurements we calculated the phase diagram for the ternary system as shown in Fig. 3 ▸. It can be seen that in a wide range of compositions the melt crystallizes directly in the cubic phase with liquidus temperatures well below the value of ∼2200°C previously demonstrated to be suitable for Czochralski growth from iridium crucibles (Uecker *et al.*, 2008 ▸; Kränkel *et al.*, 2020 ▸). The lowest liquidus points below 2100°C were experimentally found for compositions close to (Sc_0.5_Y_0.5_)_2_O_3_. The *FactSage* calculations indicate a global minimum liquidus point for the ternary system of 2053°C located at the azeotrope point of the Sc_2_O_3_–Y_2_O_3_ binary system at (Sc_0.45_Y_0.55_)_2_O_3_.

**Figure 4:**
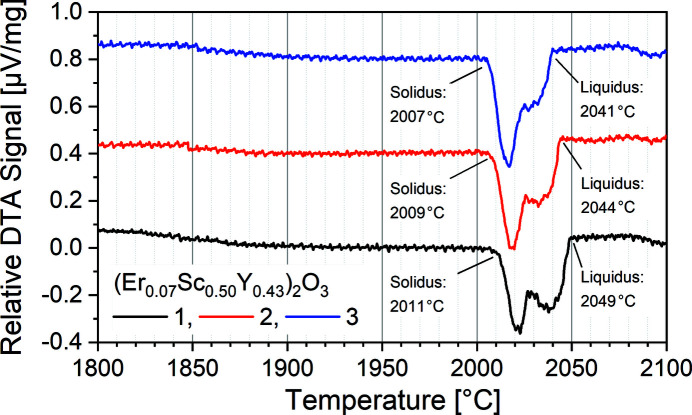
DTA curves for the composition (Er_0.07_Sc_0.50_Y_0.43_)_2_O_3_. For clarity, offsets of 0.4 µV mg^−1^ and 0.8 µV mg^−1^ were added to the second and third measurements, respectively.

To account for our target application in lasers, we replaced a fraction of the yttria by erbia, leaving us with a composition of (Er_0.07_Sc_0.5_Y_0.43_)_2_O_3_ for all further investigations. In the notation conventionally used for ion-doped laser crystals, such a crystal with 7 at.% of the host cations replaced by doping ions could be written as Er(7 at.%):(Sc_0.54_Y_0.46_)_2_O_3_. The corresponding Er-doping concentration was shown to be well suited for lasers with emission in the 3 µm range (Li *et al.*, 2012 ▸). From the phase diagram of the Er_2_O_3_–Y_2_O_3_ system shown by Zinkevich (2007 ▸), we anticipated the influence of Er_2_O_3_ on the melting behavior to be low. In fact, the DTA measurements shown in Fig. 4 ▸ very clearly reveal an onset of the phase transition at temperatures around 2010°C. The melting peak ends at temperatures slightly below 2050°C, which is in very good agreement with the values presented in Fig. 3 ▸. Taking into account the expected experimental peak width of ∼30 to 40 K detailed above, such a narrow transition peak represents almost congruent melting. The minor reduction of the melting points for the three subsequent measurements is explained by increasing order and density of the sample material and frequently observed in DTA measurements.

## 3. Crystal growth by the Czochralski method   

**Figure 5:**
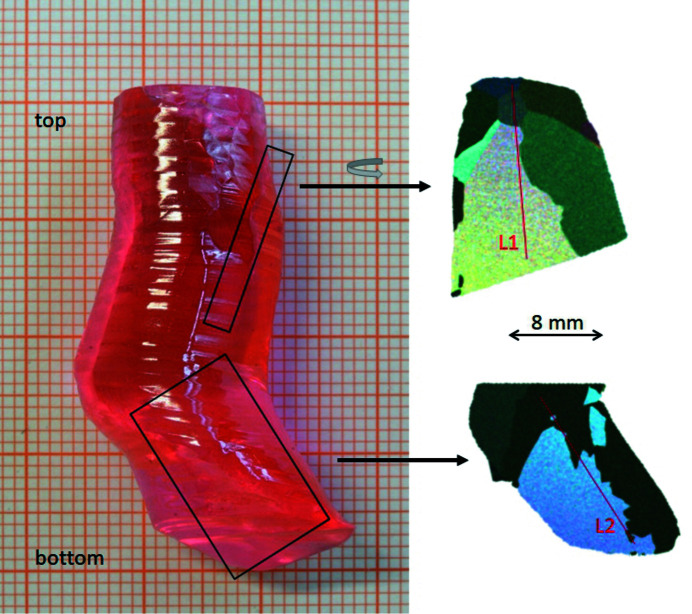
Czochralski-grown (Er_0.07_Sc_0.50_Y_0.43_)_2_O_3_. The images on the right show energy-dispersive Laue mapping images taken on longitudinal cuts from the sections indicated with black rectangles. Regions of one color indicate single crystalline parts. The red lines indicate the position of the compositional line scans in Fig. 6 ▸.

The observed reduced liquidus temperatures motivated us to investigate the feasibility of the growth of a mixed sesquioxide crystal of the composition (Er_0.07_Sc_0.50_Y_0.43_)_2_O_3_ by the conventional Czochralski method. The accordingly mixed starting powders were placed in an inductively heated iridium crucible (about 40 mm in diameter and height) embedded in ZrO_2_ and Al_2_O_3_ insulation. The growth was seeded by a 5 mm-thick iridium rod. It took place under Ar atmosphere at a growth rate of 0.7 mm h^−1^ and a rotation rate of 10 rpm using automated diameter control. An active Ir afterheater was utilized to reduce the thermal gradients, as this concept has been successfully applied to other high-temperature growth processes from Ir crucibles (Uecker *et al.*, 2008 ▸). The as-grown crystal is shown in Fig. 5 ▸. It is clear and transparent and shows facets in parts of its surface. The diameter varies between 12 and 18 mm at a length of about 40 mm. The length was limited by the eccentric shape of the lower part of the crystal. The growth was stopped when the growing crystal touched the crucible walls. The crystal is clear and shows the expected pink color due to the Er^3+^ doping, but it also exhibits a few cracks.

The foot formation seen in the bottom part of the crystal in Fig. 5 ▸ is a known issue in the Czochralski growth of various high-melting materials (Schwabe *et al.*, 2011 ▸). We expect to avoid it for the next generation of crystals by using carefully aligned single crystalline seed crystals. As previously mentioned, sesquioxide crystals exhibit strong absorption for thermal radiation at high temperatures (Fornasiero *et al.*, 1999 ▸, 2000 ▸; Petermann *et al.*, 2002 ▸). Thus, the growth process can be further stabilized using a conical iridium baffle between the crucible and the afterheater, as routinely applied for the growth of rare-earth scandate crystals with perovskite structure (Uecker *et al.*, 2006 ▸).

## 4. Crystallographic and spectroscopic investigations   

To assess the microstructure, energy-dispersive Laue mappings (EDLMs) as detailed by Guguschev *et al.* (2015 ▸) were carried out using a Bruker M4 TORNADO spectrometer. For increased counting statistics, three consecutive continuous scans of the 20 µm X-ray excitation focus in a 30 µm grid over the samples were performed. At an integration time of 17 ms and a velocity of 1.8 mm s^−1^ we obtained the high-resolution Bragg peak mapping images shown in the right-hand part of Fig. 5 ▸. The different colors of the pattern represent Bragg peaks with different intensities and energies, indicating different crystallographic orientations of the respective grains. The as-grown crystal contains several grains and subgrains with different orientations. However, these EDLM results also indicate cm-scale single crystalline regions. An improved structural quality can be expected by the future use of oriented seed crystals.

**Figure 6:**
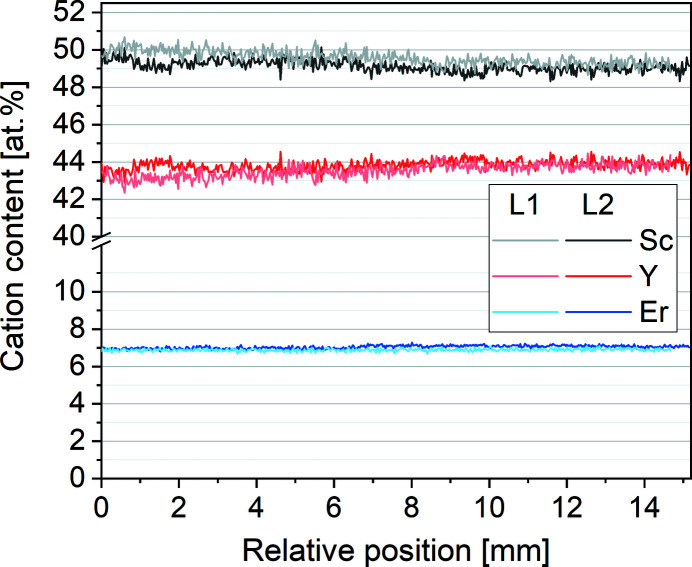
Results of the compositional analysis along the red lines L1 and L2 indicated in Fig. 5 ▸.

We further used the EDLM data to extract the micro-X-ray fluorescence (µ-XRF) elemental information by two line scans indicated with the red lines in Fig. 5 ▸. The composition along the lines was quantified by the standard-free fundamental parameter approach based on Sherman’s equation (Sherman, 1955 ▸). The results shown in Fig. 6 ▸ indicate that it is homogeneous over the whole length of the crystal and identical to the composition of the melt within the resolution of the measurement. A minimal increase of the Y content and a slight decrease of the Sc content are observable, both are below one percentage point for both samples. As shown in Fig. 5 ▸, the line scans cross several grain boundaries, and within the resolution of the measurement, no segregation at grain boundaries is observed. These results are in good agreement with the nearly congruent melting behavior shown in Fig. 4 ▸.

**Figure 7:**
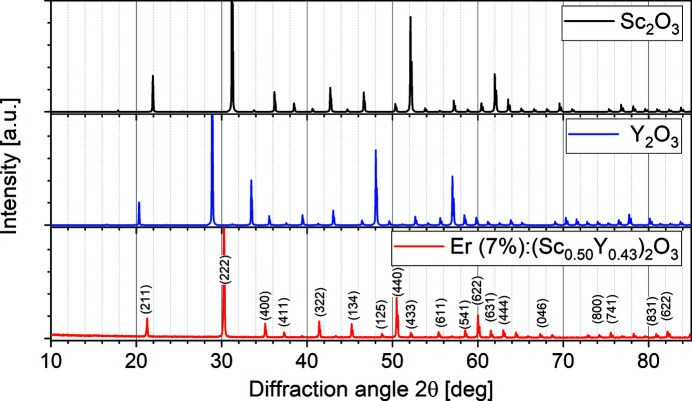
Powder diffraction pattern and Miller indices of (Er_0.07_Sc_0.50_Y_0.43_)_2_O_3_ compared to patterns calculated by the software *Fullprof* based on data (Geller *et al.*, 1967 ▸; Ferreira *et al.*, 2006 ▸) for Sc_2_O_3_ and Y_2_O_3_.

X-ray powder diffraction measurements on ground pieces confirm the cubic structure of the Czochralski-grown disordered (Er_0.07_Sc_0.50_Y_0.43_)_2_O_3_ crystal. Fig. 7 ▸ shows the calculated spectra (Jain *et al.*, 2013 ▸) for Sc_2_O_3_ (*a* = 9.846 Å; Knop & Hartley, 1968 ▸) and Y_2_O_3_ (*a* = 10.604 Å; Paton & Maslen, 1965 ▸) in comparison to the measured data for the mixed crystal. As expected, the measured lines for the mixed crystal are found at angles between those for the constituents. The spectrum resembles all features of the constituents’ spectra, and all peaks can be assigned to known lattice planes. This clearly indicates the cubic bixbyite structure ( 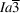 ) of our mixed sesquioxide crystal. A short Rietveld refinement using the software *Fullprof* (https://www.ill.eu/sites/fullprof/) yielded a lattice parameter of 10.205 (5) Å. This value is in reasonable agreement with the interpolated value of 10.221 Å obtained by Vegard’s law (Knop & Hartley, 2011 ▸; Paton & Maslen, 1965 ▸; Vegard, 1921 ▸; Saiki *et al.*, 1985 ▸). A more detailed evaluation is not regarded insightful given the minor changes in the composition of this very particular mixed sesquioxide crystal (see Fig. 6 ▸).

**Figure 8:**
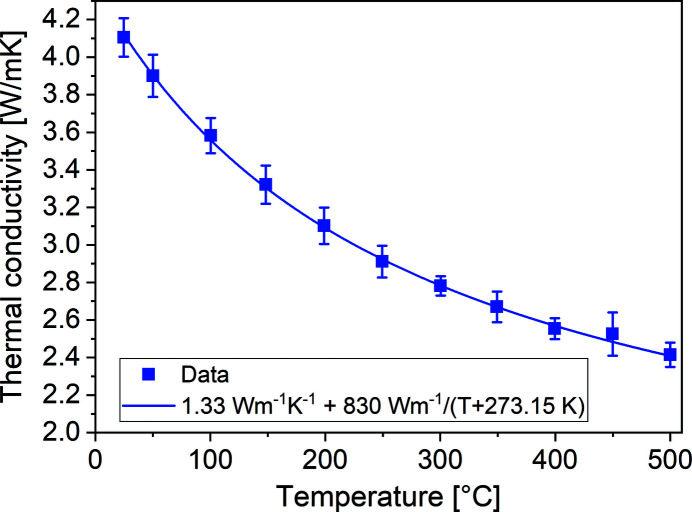
Temperature-dependent thermal conductivity of (Er_0.07_Sc_0.50_Y_0.43_)_2_O_3_.

Cubic rare-earth sesquioxides are known to exhibit a comparably high thermal conductivity among oxide crystals, which is important for high-power laser operation. However, the disordered nature of the mixed sesquioxides and the high Er-doping concentration in combination with the large mass differences between Er, Sc and Y are expected to cause a significant reduction of the thermal conductivity (Peters *et al.*, 2011 ▸; Peters *et al.*, 2009 ▸). To investigate the thermal conductivity of our crystal, we measured the thermal diffusivity in the temperature range between room temperature and 500°C by the laser flash technique (Parker *et al.*, 1961 ▸) using a NETZSCH LFA427 apparatus. The measurement was repeated three times for each temperature and the whole measurement was repeated at four different spots on the sample. By multiplication with the calculated density of 4.82 g cm^−3^ resulting from the measured lattice parameter and the temperature-dependent heat capacity calculated using *FactSage* (GTT Technologies, 2020 ▸) assuming an ideal solid solution, we obtained the thermal conductivity values plotted in Fig. 8 ▸. The room-temperature value amounts to 4.1 W m^−1^ K^−1^, which is in reasonable agreement with the values reported by Peters *et al.* (2011 ▸, 2009 ▸) for Yb-doped LuScO_3_ mixed sesquioxide crystals.

**Figure 9:**
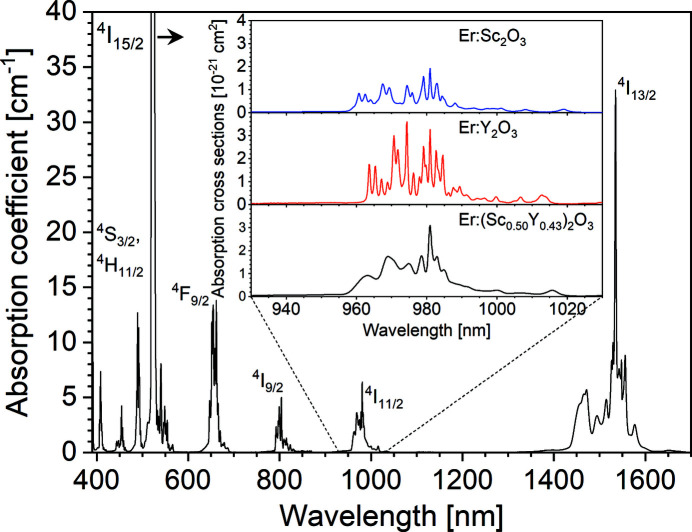
Ground state absorption coefficients of (Er_0.07_Sc_0.50_Y_0.43_)_2_O_3_ in the visible and near infrared spectra range. The labels at the absorption bands denote the name of the Stark multiplet responsible for the absorption from the ground state ^4^ *I*_15/2_. The inset shows the cross sections of the ^4^ *I*_15/2_ → ^4^ *I*_11/2_ ground state absorption of Er^3+^ in (Er_0.07_Sc_0.50_Y_0.43_)_2_O_3_ in comparison to the absorption of Er^3+^ in Sc_2_O_3_ and Y_2_O_3_.

Finally, to investigate the optical properties of the crystal, we performed optical transmission measurements in the visible and near-infrared spectral range. The measurements were performed in a Perkin Elmer Lambda 1050 UV/Vis/NIR spectrophotometer set to a resolution of 0.4 nm, to ensure the narrowest absorption features were fully resolved. By using the Beer–Lambert law we obtained the absorption coefficients shown in Fig. 9 ▸. We could assign all absorption bands to the known energy levels of Er^3+^. The absorption into the levels ^4^H_11/2_, ^4^S_3/2_ and ^4^F_9/2_ located in the visible is responsible for the pink color of the crystal, characteristic for Er-doped materials. To further confirm the disordered structure of the mixed crystal, we compared the absorption characteristics in the wavelength range around 1 µm corresponding to absorption from the ground state ^4^ *I*_15/2_ into the excited state ^4^ *I*_11/2_ of the Er^3+^ ions with those of Er-doped yttria and scandia. This transition represents the typical pump wavelength for Er^3+^ lasers emitting at 1.6 µm or 3 µm and is thus of particular relevance for our planned application as a gain medium in solid-state lasers. By division of the absorption coefficient by the calculated Er^3+^ ion density of the melt composition of 2.11 × 10^21^ cm^−3^, we obtained the absorption cross sections shown in the inset of Fig. 9 ▸. It can be clearly seen that the absorption features of the mixed crystal are broadened compared to the pure Er^3+^-doped sesquioxides. This is caused by an inhomogeneous line broadening induced by the disordered crystal structure of the mixed crystal with a statistical distribution of the cations. It should, however, be noted that in this particular case the broadening does not lead to a significant reduction of the peak absorption cross section, as by coincidence the strong absorption line around 981 nm is found at the same position in both constituents and thus not strongly broadened. Nevertheless, despite the comparably high peak absorption cross section of 3.1 × 10^−21^ cm^2^ at this wavelength, excited state absorption (ESA) effects may make other pump wavelengths more efficient in future laser experiments (Li *et al.*, 2012 ▸) and further investigations are required to fully explore the potential of Czochralski grown (Er_0.07_Sc_0.50_Y_0.43_)_2_O_3_, *i.e.* Er(7 at.%):(Sc_0.54_Y_0.46_)_2_O_3_, for mid-infrared lasers.

## 5. Conclusion   

In conclusion, in detailed thermodynamic investigations we have shown that in the Sc_2_O_3_–Y_2_O_3_ binary system as well as in the ternary phase diagram between Lu_2_O_3_, Sc_2_O_3_ and Y_2_O_3_, a significant range of compositions with melting temperatures below 2200°C exists, which facilitates crystal growth by the conventional Czochralski method. For the composition (Er_0.07_Sc_0.50_Y_0.43_)_2_O_3_ the melting point is found at temperatures below 2050°C. This enabled for the first time, to the best of our knowledge, the growth of a high-quality, large size mixed sesquioxide crystal by the Czochralski method from an iridium crucible. Due to the lack of a suitable seed crystal, this first crystal contains several grains and subgrains and suffers from an eccentric foot formation during the growth. However, these issues can be solved by techniques routinely applied to the growth of high melting point oxide crystals.

Powder diffraction measurements confirm the cubic bixbyite structure of (Er_0.07_Sc_0.50_Y_0.43_)_2_O_3_ and EDLM measurements reveal large single crystalline areas. The segregation coefficients for all cations are close to unity for this composition and the room-temperature thermal conductivity amounts to 4.1 W m^−1^ K^−1^, which is higher than for other materials used for ultrafast laser applications (Südmeyer *et al.*, 2009 ▸). In future experiments we will investigate further laser relevant properties of this crystal such as fluorescence dynamics, emission cross sections and ESA at possible pump wavelengths. Moreover, the quality of the crystal is appropriate to perform laser experiments on the 3 µm transition of Er^3+^.

Due to a previously reported strong line broadening, in particular mixed sesquioxide samples doped with Yb^3+^ or Tm^3+^ are suited for high average power, sub-100 fs ultrafast lasers (Baer *et al.*, 2009 ▸; Saraceno *et al.*, 2011 ▸; Südmeyer *et al.*, 2009 ▸; Stevenson *et al.*, 2018 ▸). Our results indicate that the growth of such mixed sesquioxide crystals, doped with any rare-earth ion should be possible by the Czochralski method. These results give – for the first time – a realistic perspective for a future commercial availability of high-quality mixed cubic rare-earth sesquioxide crystals.

## 6. Declaration of competing interest   

The authors declare that they have no competing financial interests or personal relationships that could have appeared to influence the work reported in this paper.

## 7. Data availability   

The datasets generated during and/or analyzed during the current study are available from the corresponding author on request.
